# Recent progress of principal techniques used in the study of Müller glia reprogramming in mice

**DOI:** 10.1186/s13619-024-00211-z

**Published:** 2024-12-12

**Authors:** Zhiyuan Yin, Jiahui Kang, Haoan Xu, Shujia Huo, Haiwei Xu

**Affiliations:** 1 Key Lab of Visual Damage and Regeneration & Restoration of Chongqing, Southwest Eye Hospital, Southwest Hospital, Third Military Medical University (Army Medical University), Chongqing, 400038 P.R. China; 2 School of Life Sciences and Technology, Tongji University, Shanghai, 200092 China

**Keywords:** Müller glia, Reprogramming, Retina, Mice, Multi-omics, Technique

## Abstract

In zebrafish, Müller glia (MG) cells retain the ability to proliferate and de-differentiate into retinal progenitor-like cells, subsequently differentiating into retinal neurons that can replace those damaged or lost due to retinal injury. In contrast, the reprogramming potential of MG in mammals has been lost, with these cells typically responding to retinal damage through gliosis. Considerable efforts have been dedicated to achieving the reprogramming of MG cells in mammals. Notably, significant advancements have been achieved in reprogramming MG cells in mice employing various methodologies. At the same time, some inevitable challenges have hindered identifying accurate MG cell reprogramming rather than the illusion, let alone improving the reprogramming efficiency and maturity of daughter cells. Recently, several strategies, including lineage tracking, multi-omics techniques, and functional analysis, have been developed to investigate the MG reprogramming process in mice. This review summarizes both the advantages and limitations of these novel strategies for analyzing MG reprogramming in mice, offering insights into enhancing the reliability and efficiency of MG reprogramming.

**Graphical Abstract:**

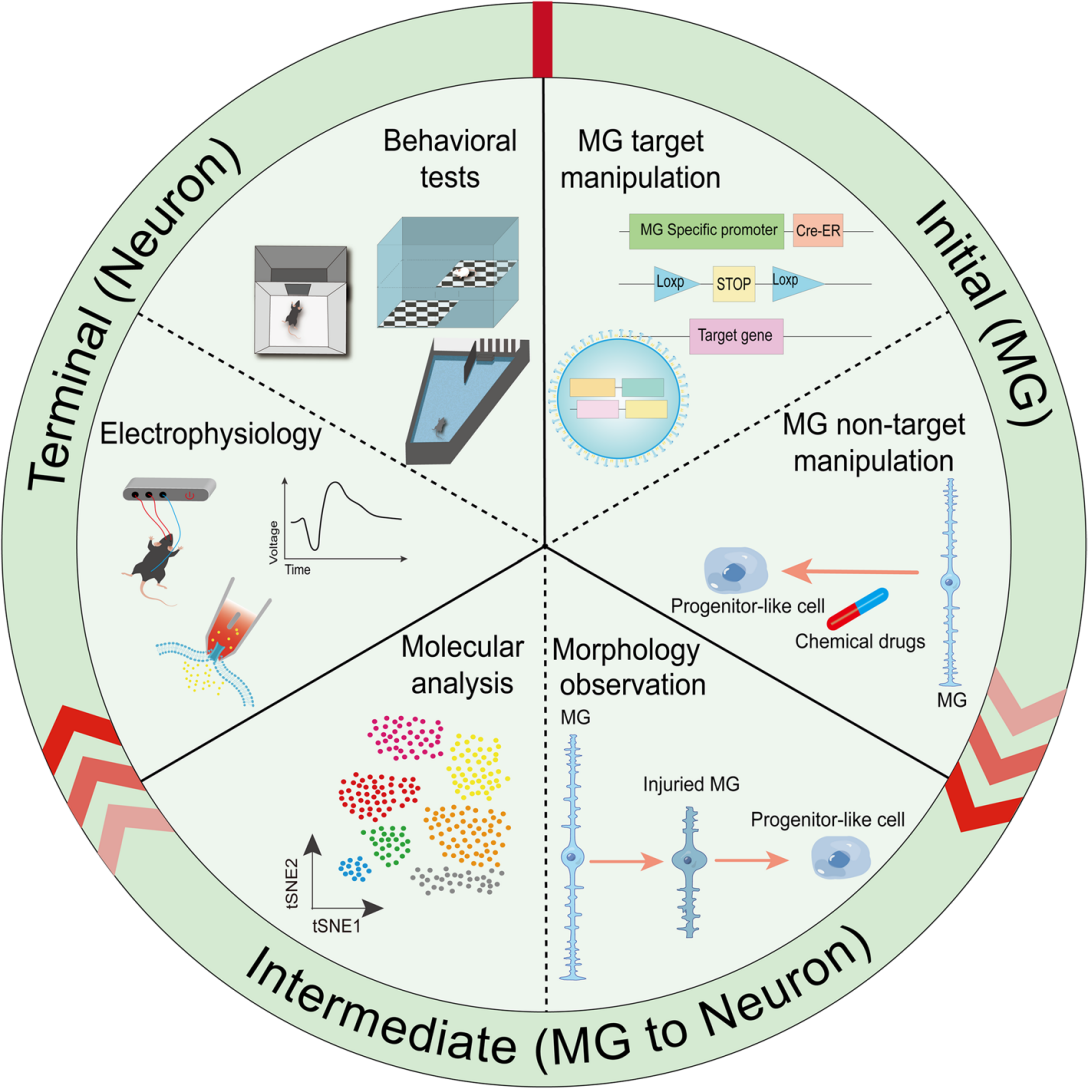

## Background

Injury, degeneration, and neuronal loss in the retina are major contributors to permanent vision impairment and blindness. Retinal degenerative diseases, such as age-related macular degeneration, retinitis pigmentosa, diabetic retinopathies, glaucoma, and numerous hereditary retinal disorders (Wan and Goldman 2016), currently lack a cure. A crucial therapeutic strategy for retinal degeneration involves either safeguarding the compromised neurons or substituting the deceased ones with grafted or newly-generated neurons. Gene therapy, stem cell transplantation, glial cell reprogramming, and retinal prostheses are proposed as potential remedies for these conditions (Santos-Ferreira et al. 2017; Pavlou and Reh 2023). Cell reprogramming for retinogenesis emerges as a promising technique for vision restoration. This approach effectively bypasses ethical issues, minimizes risks such as tumor formation or immune rejection, and offers sustained benefits for the retina (Jin et al. 2019; Hoang et al. 2020).

Müller glia (MG) are optimal candidates for reprogramming to regenerate the injured or degenerative retina. In mammals, MG typically trigger a gliotic reaction in response to retinal injury (Hippert et al. 2015; Thomas et al. 2016), whereas in zebrafish, this response is brief and coupled with regeneration. In zebrafish, retinogenesis pathways in MG prompt their de-differentiation, followed by interkinetic nuclear migration and asymmetric division, resulting in multipotent MG-derived progenitors. These progenitors further divide, migrate across retinal layers, and differentiate into diverse retinal neurons, except rods (Chohan et al. 2017). Conversely, mammalian MG tend to undergo an initial gliotic surge and rapidly revert to a quiescent state, exhibiting fibrosis and glial scarring instead of reprogramming potential (Bringmann et al. 2009).

Direct reprogramming has successfully converted somatic cells, including astrocytes, fibroblasts, and MG cells, into neurons (Li et al. 2019; Yamada et al. 2023). Inspired by MG reprogramming in zebrafish and somatic cell conversion in mammals, notable advancements have been made in MG reprogramming in mice over the past decade. Transcription factors regulating retinal development and cell identity are extensively employed for MG reprogramming. Co-expression of transcription factors like Pou4f2 (Brn3b), Atoh7 (Math5), Islet1, and Ascl1 has effectively transformed MG cells into ganglion cells (Xiao et al. 2021; Todd et al. 2022). Ptbp1 knockdown or MAP4K inhibition has also prompted MG reprogramming into ganglion cells (Zhang et al. 2023; Zhou et al. 2020). Overexpressing Ascl1, primarily found in retinal progenitor cells, facilitated MG transformation into amacrine and bipolar cells (Jorstad et al. 2017). Similarly, NFI knock-down, which dictates cell-cycle exit and late-born retinal cell generation, resulted in MG conversion to amacrine-like and bipolar-like cells (Hoang et al. 2020). For photoreceptors, differentiation from glia to rods (Yao et al. 2018) or cone-like cells (Pinsonneault et al. 2023) was achieved by co-expressing β-catenin with transcription factors (Otx2, Crx, and Nrl) or Ikzf1 and Ikzf4, respectively. NeuroD1, known to reprogram brain astrocytes into neurons in various disease models (Guo et al. 2014; Wu et al. 2020; Chen et al. 2020; Ge et al. 2020), has been observed in photoreceptor (Ochocinska et al. 2012) and amacrine cell development (Cho et al. 2007). Overexpression of NeuroD1 in MG resulted in their conversion to amacrine, ganglion, photoreceptor, and horizontal cells using dual adeno-associated viruses (AAV) (Xu et al. 2023). Nonetheless, traditional reprogramming methods often face skepticism. Xie et al. demonstrated that novel AAV-based tools could curb neuronal leakage and found that NeuroD1 could not indeed convert MG cells into neurons (Xie et al. 2023). Additionally, rigorous investigations showed that Ptbp1 knockout or knockdown failed to reprogram MG cells into neurons (Wang and Zhang 2023; Xie et al. 2022; Hoang et al. 2022).

Thus, effective and precise MG cell manipulation techniques and validation methods are crucial to prevent misconceptions. Advances in gene editing technology are integral to MG reprogramming development. Recently, researchers have refined MG reprogramming processes to include morphological verification of MG-derived neurons, assessments of gene transcriptional activity, epigenetic changes, and cellular functionality (Hoang et al. 2020; Xiao et al. 2021; Todd et al. 2022; Zhou et al. 2020; Jorstad et al. 2017, 2020; Yao et al. 2018; Pinsonneault et al. 2023; Sanges et al. 2016; Pesaresi et al. 2018). These studies extensively used tools like global and conditional gene-deletion animal models (e.g., Cre-Loxp system), AAV, CRISPR-cas9 system, single-cell RNA sequencing (scRNA-seq), and Assay for Targeting Accessible-Chromatin sequencing (ATAC-seq). Despite significant progress, reprogramming protocols still heavily depend on trial-and-error techniques. Novel predictive tools may provide viable solutions to these challenges. This review focuses on key tools for investigating MG reprogramming, examining related processes such as MG manipulation in the early stages, morphological observations, molecular analysis during intermediate stages, and vision restoration assessments in later stages.

### MG-targeted manipulation used at the initial stage of MG cell reprogramming

MG-targeted manipulations directly regulate gene expression in MG cells using MG-specific promoters. Currently, techniques such as AAV and Cre-Loxp systems enable either overexpression or knockdown of specific transcription factors (e.g., Ascl1, NeuloloroD1, Otx2, Crx, Nrl, Pou4f2, Atoh7, Islet1, Ikzf1, Ikzf4, Ptbp1) within MG cells (Tables 1 and 2).

**Table 1:** Transgenic mouse lines and viral vectors for Müller target

Lines or virus	Promoter	Specificity	Efficiency	Reference
Gfap-Cre/ERT2	Gfap	Moderate/ Low	High	Fu et al. 2020
Slc1a3-Cre/ERT2	Slc1a3	Very high	Very high	Xiao et al. 2021; Jorstad et al. 2017; Pinsonneault et al. 2023; Xie et al. 2023; Hoang et al. 2022; Todd et al. 2021
Rlbp-Cre/ERT2	Rlbp	High	High	Jorstad et al. 2017
AAV1	Gfap	Very high	Low	Gao et al. 2022
AAV2	Gfap	Low	High	Fu et al. 2020; Gao et al. 2022; Bonilla-Pons et al. 2022
AAV5	Gfap	Very high	Low	Xie et al. 2023; Gao et al. 2022
AAV6 (ShH10)	Gfap	Moderate	Moderate	Xiao et al. 2021; Xie et al. 2022, 2023; Gao et al. 2022; Bonilla-Pons et al. 2022; Hamon et al. 2019
AAV7	Gfap	Moderate	Moderate	Xu et al. 2023
AAV8	Gfap	Moderate	High	Fu et al. 2020; Gao et al. 2022; Bonilla-Pons et al. 2022
AAV9	Gfap	High	High	Xiao et al. 2021; Xu et al. 2023; Gao et al. 2022
PHP.eB	Gfap	High	High	Zhou et al. 2020; Xie et al. 2023

#### AAV-mediated manipulation of MG

AAV is a widely used gene delivery platform due to its targeted efficiency and high safety profile. Various AAV serotypes have been utilized in MG reprogramming studies (Table 1). The AAV-ShH10 serotype, in particular, is favored for gene delivery to MG because of its selectivity. AAV-mediated gene manipulation offers a rapid and straightforward method for inducing MG reprogramming in murine models.

Yao et al. used AAV to deliver β-catenin, Otx2, Crx, and Nrl, promoting MG proliferation and rod differentiation in Gnat1^rd17^Gnat2^cpfl3^ double mutant mice (Yao et al. 2018). Similarly, Xiao et al. established an AAV-based co-expression system for Math5 and Brn3 in reporter-labeled RGCs derived from MG in Brn3b^AP/AP^ knockout mice (Xiao et al. 2021). Notably, MG were differentiated into various cell types through NeuroD1 ectopic expression using two different viral vectors. AAV7m8 GFAP681::GFP-NeuroD1 transformed MG into inner retinal neurons such as RGCs and amacrine cells, whereas AAV9-GFAP104::NeuroD1-GFP was more effective in producing horizontal cells and other outer retinal neurons. NeuroD1’s capacity to induce MG to transform into rod photoreceptors is dose-dependent; 10^11^ GC/mL of AAV9-GFAP104::NeuroD1-GFP prompted migration of some GFP^+^ cells to the outer nuclear layer, with conversion efficiency nearing that of higher doses (Xu et al. 2023).

**Fig. 1:**
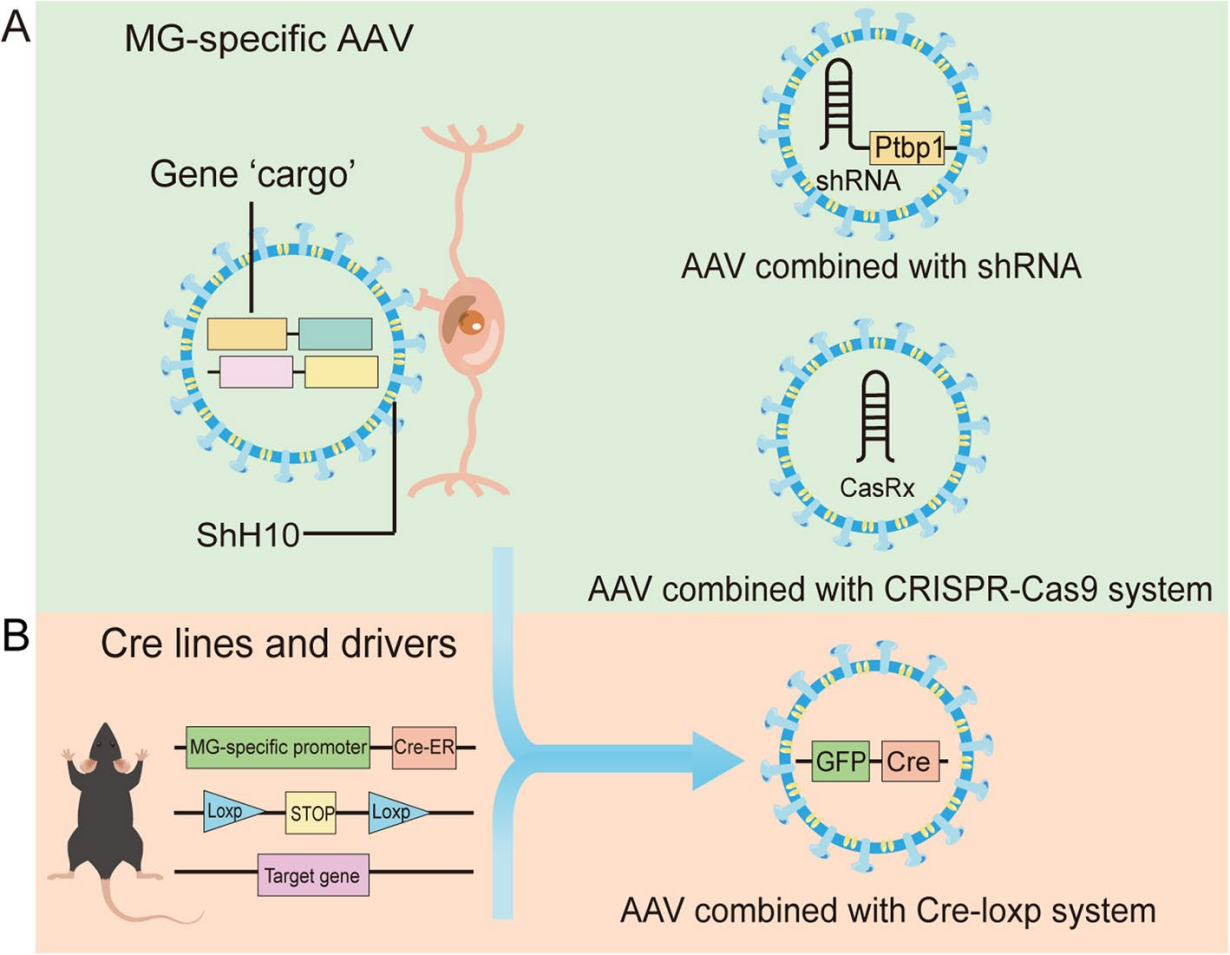
The tools targeting and manipulating MG for reprogramming. **A** AAV6 (ShH10) specifically targets MG as a gene delivery platform and carries shRNA or CRISPR components for specific gene knock-down in mice. **B** General structure of Cre ^ERT2^-Loxp system, AAV carries the Cre enzyme to manipulate Loxp system in mice

Combining AAV with shRNA or the CRISPR system enables precise and efficient target gene knockdown (Karimian et al. 2019; Evers et al. 2016). shRNA, produced by a stem-loop structure, can be introduced into cells to disrupt gene function (Rao et al. 2009). AAV-shRNA-mediated RhoA knockdown has been shown to significantly enhance axonal regeneration and optic nerve survival in rats (Koch et al. 2014). Similarly, AAV-modified Ptbp1 knockdown effectively reprogrammed astrocytes and facilitated MG neuronal reprogramming (Qian et al. 2020; Fu et al. 2020; Fig. 1A). However, shRNA knockdown can suffer from efficiency variability and potential off-target effects (Fu 2023).

The CRISPR-Cas system emerges as an advantageous alternative for gene knockout. It functions as an essential bacterial defense mechanism against foreign DNA, consisting of small guide RNA (sgRNA) and Cas protein, which enable precise genome editing. Although AAV’s 5 kb packaging capacity can limit its application, a dual-AAV approach, with separate vectors for Cas9-encoding DNA and sgRNA, can overcome this constraint (Yang et al. 2016). Transforming MG into RGCs in both healthy and damaged retinas has been achieved using AAV expression of Cas13d and aligned guide RNAs targeting Ptbp1 mRNA (Zhou et al. 2020). Conversely, AAV-GFAP-CasRx-Ptbp1 does not significantly suppress Ptbp1 in astrocytes (Wang et al. 2021). Xie et al. noted only marginal suppression of Ptbp1 at mRNA and protein levels using CRISPR-CasRx in MG (Xie et al. 2022; Fig. 1A). Hoang et al. successfully knocked out Ptbp1 from MG cells via the Cre-Loxp system (Hoang et al. 2022).

Compared with gene knockout, shRNA knockdown may lead to false negatives due to gene compensation within the organism. Hoang et al. observed Ptbp2 upregulation following Ptbp1 knockout, suggesting functional compensation (Vuong et al. 2016). This provides insight into the discrepancies in Ptbp1 knockdown and knockout outcomes and the contentious role of Ptbp1 in MG cell reprogramming. Rossi et al. demonstrated that zebrafish egfl7 gene mutations or knockdown lead to variations in vascular phenotype. Genetic mutation in egfl7 resulted in less severe vascular defects due to extracellular matrix gene upregulation, lacking in gene knockdown scenarios (Rossi et al. 2015).

AAV gene transfer specificity is influenced by promoter selection, adjacent cDNA sequences, virus concentration, and post-infection timing (Wang et al. 2021; Le et al. 2022; Martin and Poche 2019). High AAV titers (1–2 × 10^13^ GC/mL) pose risks like tissue damage and neuronal leakage at 1 µl volumes, leading to neuronal de-differentiation, which undermines experimental reliability (Xiang et al. 2021). Glial cells are similarly vulnerable, necessitating careful dose selection to preclude adverse effects. While the GFAP promoter demonstrates high initial activity in AAV-infected astrocytes, a subsequent decline may necessitate increased doses for complete fate transitions (Fu 2023). Efficient enhancer binding may alleviate these issues (Xu et al. 2022).

Intra-retinal AAV injections may provoke immune or inflammatory responses (Bucher et al. 2021), impacting MG cell reprogramming. The NF-κB pathway plays a critical role in innate immunity (Yu et al. 2020), while its activation aids MG reprogramming, sustained activation hinders proliferation in avian and murine models (Palazzo et al. 2020, 2022). AAV capsid interaction with toll/interleukin-1 receptor 2 triggers NF-κB, inducing inflammatory cytokine production in primary human cells (Hösel et al. 2012).

AAV-induced retinal immune responses are contingent on factors like AAV dose, injection site, DNA-to-capsid ratio, promoter sequence, and transgenic protein type (Bucher et al. 2021). Preclinical findings indicate dose-dependent immune reactions to AAV gene therapy, with dosages above 1 × 10^11^ GC/eye provoking ocular responses, absent below this threshold (Ramachandran et al. 2017; Tobias et al. 2019; Dufour et al. 2020; Ghazi et al. 2016). Employing non-toxic promoter sequences, maintaining optimal DNA-to-capsid ratios, and preferring sub-retinal AAV injections may diminish immune responses and local inflammation. Engineering carrier capsids with low immunogenicity to target MG cells can further reduce this risk (Bucher et al. 2021).

Addressing the challenges of long-term AAV retinal injections necessitates evaluating the infection duration and efficacy within MG cells and its impact on MG reprogramming.

#### Cre-loxp system-mediated manipulation of MG

The Cre-Loxp system, coupled with tamoxifen induction (Cre/ERT2 system), is a powerful tool for MG-specific and temporally controlled manipulations (Branda and Dymecki 2004; Fig. 1B). The Cre enzyme facilitates genetic modifications like deletion, inversion, and translocation between Loxp sites, directed by cell-specific promoters. Adding a human estrogen receptor (ER) ligand to Cre allows cytoplasmic localization, with tamoxifen binding prompting nuclear entry and Loxp site cleavage, enabling precise temporal control of gene recombination (Feil et al. 1996).

Numerous Cre-dependent transgenic mouse lines leverage MG-specific promoters like GFAP (Yao et al. 2018; Fu et al. 2020), Slc1a3 (Glast) (Xiao et al. 2021; Jorstad et al. 2017; Pinsonneault et al. 2023; Xie et al. 2023; Hoang et al. 2022; Todd et al. 2021), and Rlbp (Jorstad et al. 2017; Table 1). The tamoxifen-inducible Slc1a3-Cre^ERT2^ line is prominent, with Slc1a3 being a major cerebellar glutamate transporter (Takatsuru et al. 2007), also present in the inner ear (Takumi 1997) and retina (Derouiche 1996). In retinal applications, Slc1a3-Cre-induced tdTomato expression is MG-specific, warranting further validation to exclude other retinal cell types overlap, such as RGCs and photoreceptors.

The Cre-Loxp recombination system is versatile for conditional gene expression and lineage tracing, offering targeted chromosomal DNA modifications (Nagy 2000). For instance, Blackshaw et al. used tamoxifen-fed Slc1a3-Cre^ER^; CAG-lsl-Sun1-GFP mice crossed with Nfia/b/x-Loxp lines to delete NFI from MG, increasing photoreceptor/bipolar markers Crx and amacrine/ganglion/horizontal cell markers HuC/D and NeuN (Hoang et al. 2020).

Tetracycline-inducible transgenic models enable precise temporal gene overexpression. Slc1a3-Cre^ER^: LNL-tTA: teto-mAscl1-GFP mice were crossed with tetO-Pou4f2-tetO-Islet1 lines, upon Cre and tetracycline induction, facilitate Ascl1, Pou4f2, and Islet1 expression (Todd et al. 2022).

Although lineage-based MG manipulation proves reliable, its translation to primate models presents challenges.

#### Cre-loxp system combined with AAV to manipulate MG

The Cre-Loxp system, when integrated with AAVRGC technology, represents a fundamental strategy for managing gene expression critical to MG reprogramming (Qian et al. 2020) (Fig. 1B). For example, Zhou et al. employed AAV-GFAP-GFP-Cre to selectively trigger tdTomato expression in MG within Ai9 (Rosa26-LSL-tdTomato) mice (Zhou et al. 2020). Nonetheless, this approach can result in unintended low-level expression of fluorescent proteins in neurons (Xie et al. 2022). In a related context, Math5 and Brn3b were introduced into mouse MG via an AAV vector carrying Cre sites to convert MG into retinal ganglion cells (RGCs) (Xiao et al. 2021). However, due to pervasive neuronal leakage, Chen et al. suggested that AAV-based Cre recombination might be inadequate for assessing MG-to-RGC transformation driven by NeuroD1 overexpression. Attempts to mitigate transgene-dependent leakage by reducing AAV dosage, utilizing different AAV serotypes, or exploring alternative injection methods proved ineffective. Moreover, if AAV’s cellular tropism shifts from glial cells to neurons, reprogramming efficiency is typically significantly compromised (Xie et al. 2023). To overcome these challenges, a novel AAV-GFAP-based tool employing reprogramming factors located downstream of a reporter gene, facilitated by P2A self-cleaving sequences or CAG-FLEX dual systems, has been developed for precise MG reprogramming (Xie et al. 2023). Therefore, it is crucial to perform Cre immunofluorescence on a control group to ensure that any labeling detected with the Cre reporter is not a result of AAV expression leaking into neurons or from transfer by adjacent cells. Considering the current uncertainty regarding material exchange between retinal cells, fluorescence data from Cre or Loxp reporters should not be solely relied upon as definitive indicators of cell lineage (Boudreau-Pinsonneault and Cayouette 2018).

Notably, Cre-Loxp recombination in lineage-traced astrocytes might present a greater obstacle for cell conversion than encountered in wild-type mice (Chen 2021). Additional research is necessary to determine if lineage-traced mice will face comparable issues in MG reprogramming within the retina. Additionally, tamoxifen-induced toxicity may increase barriers to neuronal reprogramming, complicating lineage tracking of the reprogrammed neurons. Consequently, the AAV delivery was delayed until one to two weeks after tamoxifen induction to provide sufficient recovery and reduce the reprogramming barriers (Zhang et al. 2022; Tai et al. 2021).

In sum, AAV and Cre-Loxp mouse lines are invaluable in MG reprogramming research. AAV serves as a gene vector with clinical application potential, ensuring efficient and safe gene manipulation. However, reports on AAV manipulation in MG cells, especially concerning ptbp1 knockout or knockdown and neurod1 overexpression, have faced scrutiny (Zhou H 2020; Xie et al. 2023; Xie et al. 2022; Hoang et al. 2022; Wang et al. 2021; Qian et al. 2020). AAV-GFAP-mediated leakage expression limits its suitability for detecting MG reprogramming (Xie et al. 2022; Wang et al. 2021), as the GFAP promoter’s proximity to transgenes such as Ascl1, NeuroD1, Math5, and Cre results in varying leakage expression levels (Xie et al. 2022; Wang et al. 2021). This leakage might stem from potential cis-regulatory interactions between the GFAP promoter and transgenes. To address this, researchers strategically positioned the non-leaky fluorescent reporter gene mCherry upstream of the reprogramming factor, which reduced unwanted expression in endogenous neurons, although not entirely eliminating it (Xie and Chen 2024; Xie et al. 2023). A significant modification by Gao et al. involved removing the WPRE element from the AAV vector hGFAP-Cre-WPRE, efficiently and specifically labeling MG in Ai9 transgenic mice without significant leakage in RGCs (Gao et al. 2022). Future experiments might replace the GFAP promoter with Slc1a3 or Rlbp1 to determine if these changes effectively eliminate AAV leakage expression in neuronal cells. Accordingly, inducible Cre mouse lines, in conjunction with MG fate-tracing reporter mouse lines, are effective in labeling glial cells prior to AAV infection. For examining the GFAP promoter’s role in enabling neuronal transgenic expression within AAV-mediated systems, Slc1a3-CreER or Rlbp1-CreER lines are preferable. Moreover, Ai9 may exhibit minimal tdTomato expression before Cre recombinase activation (Xie and Chen 2022), making CAG-LSL-Sun1-GFP or R26R-EYFP more desirable. Since AAV is more transferrable to clinical applications than the Cre-Loxp system, the optimal strategy for MG cell reprogramming research is to initially use the Cre-Loxp system to identify transcription factors facilitating reprogramming, followed by precise AAV application for intraretinal therapy.

### MG non-targeted manipulation used in MG reprogramming

Directly manipulating MG presents a viable approach for gene regulation, considering cell fate conversion mechanisms. However, numerous alternative non-targeted regulatory methods exist for MG reprogramming.

Non-targeted MG regulation bypasses direct gene manipulation, offering advantages such as high regulatory efficiency, extensive options for MG reprogramming, the capability to administer multiple drugs, and the adaptability to combine multiple strategies for clinical applications. Non-targeted regulation primarily involves chemical drugs, presenting promising research avenues. Agonists or inhibitors frequently contribute to epigenetic remodeling (Jorstad et al. 2017; Hirai and Kikyo 2014), immune regulation (White et al. 2017; Conedera et al. 2019), and metabolic modulation (Gascon et al. 2016) by modulating specific target proteins or signaling pathways. For example, our previous findings demonstrated that inhibitors of MAP4K4/6/7 led to mouse MG reentering the cell cycle and resembled retinal progenitor cells following NMDA-induced retinal damage. With inhibitor withdrawal, MG exhibited amacrine or retinal ganglion cell markers (Zhang et al. 2023). It’s crucial to consider the challenges of small molecule penetration into the retina due to the blood-retinal barrier and potential off-target effects (Zhang et al. 2023).

Recently, novel non-targeted approaches for MG include cell-cell fusion and cellular interactions. Hematopoietic stem and progenitor cells or bone marrow stem cells were transplanted into a mouse retina to reprogram MG into photoreceptor progenitor, ganglion, and amacrine cells (Sanges et al. 2016; Pesaresi et al. 2018). Our recent study showed that bone marrow stem cells transferred mitochondria into MG, reducing oxidative stress and gliosis by altering MG metabolism (Huang et al. 2024). Microglia, key immune regulators in the retina, are demonstrated to influence MG reprogramming through specific subtypes in zebrafish and mice (White et al. 2017; Conedera et al. 2019; Cheng et al. 2023). Additionally, microglia/macrophages and vascular endothelial cells regulate MG reprogramming and proliferation via the VEGF and Notch signaling pathways in zebrafish (Mitra et al. 2022). Administering PLX3397 can deplete microglia, and withdrawing PLX3397 induces repopulated microglia to promote MG conversion into retinal precursor cells in mice (Cheng et al. 2023).

Furthermore, exogenous substances like exosomes and mitochondria also possess MG reprogramming potential (Fridman et al. 2022; Nascimento-Dos-Santos et al. 2020; Wu et al. 2022; Zhang et al. 2021). Extracellular vesicles and mitochondria act as specialized carriers for biomolecules, including metabolites and miRNA. Mitochondria-to-nucleus signaling also mediates the chemical reprogramming process (Mahato et al. 2020).

### Using lineage tracing to display the intermediate state of MG cell reprogramming

Both MG-targeted and non-targeted approaches present unique advantages in manipulating MG cells. Although AAV-mediated tools show high specificity for MG cells, completely avoiding neuronal leakage remains a challenge. Thus, rigorous lineage tracing, particularly employing the Cre-Loxp system, is crucial for ensuring the accuracy of MG reprogramming (Wang and Zhang 2022).

#### Observation of MG morphology changes during the reprogramming process

MG undergoes significant morphological changes as it transitions into neurons. A key aim of evaluating MG reprogramming is precisely identifying and differentiating the morphological traits at each stage.

In the undamaged mouse retina, MG microvillus outer processes are distributed across the outer nuclear layer, while the vitreal endfeet are concentrated in the ganglion cell layer (Wang et al. 2017a, b). Following acute damage, MG experiences hypertrophy and increased stiffness, ultimately leading to gliosis. It then loses its glial form, adopts a progenitor-like state, and produces progeny cells that migrate to sites of previous neuron loss (Lourenco et al. 2021; Lahne et al. 2020).

**Fig. 2:**
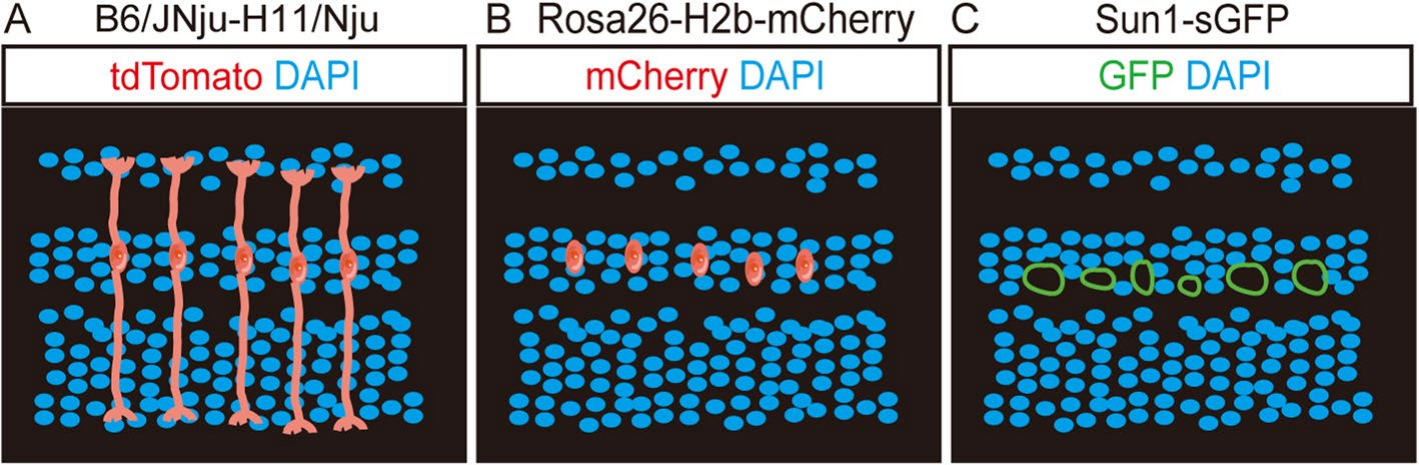
The tools used for MG morphology observation during the reprogramming process. **A** Visualizing exquisite MG morphology by B6/JNju-H^11em1Cin^ reporter mice. **B** Visualizing MG nuclei morphology by Rosa26-H2b-mCherry reporter mice. **C** Visualizing MG nuclei membrane by Sun1-sGFP reporter mice

Recently, single-cell morphological labeling has been achieved using MG-specific promoters and cytosolic fluorescent proteins like GFP, mCherry, and tdTomato, facilitated through viruses or transgenic mouse lines. It is crucial to evaluate expression across multiple mouse strains to appropriately limit Cre recombinase expression. Previous research, along with our findings, demonstrated that certain reporter mice, such as Rosa26-LSL-tdTomato (Ai9) (Todd et al. 2021, 2022), Rosa26-LSL-EYFP (Pinsonneault et al. 2023), and B6/JNju-H^11em1Cin (CAG−Loxp−ZsGreen−Stop−Loxp−tdTomato)^/Nju, exhibited detailed MG morphology (Fig. 2A; Table 2). In contrast, Rosa26-H2b-mCherry mice (Fig. 2B; Table 2) label only MG nuclei, and Sun1-sGFP mice specifically label the nuclear membrane (Hoang et al. 2020, 2022; Xie et al. 2022, 2023) (Fig. 2C; Table 2).

**Table 2:** Reporter mouse lines for observation of Müller morphology

Reporters	Fluorescent protein	Protein localization	Reference
Rosa-CAG-lsl-Sun1-GFP	GFP	nuclear membrane	Hoang et al. 2020, 2022; Xie et al. 2022, 2023
Rosa26-H2b-LSL-mCherry	mCherry	nuclei	Fig. 2B
Rosa26-LSL-tdTomato (Ai9)	tdTomato	cell nuclei/membrane /cytoplasm	Todd et al. 2021, 2022
B6/Jnju-H11^em1Cin(CAG−Loxp−ZsGreen−Stop−Loxp−tdTomato)^/Nju	tdTomato	cell nuclei/membrane /cytoplasm	Fig. 2A
Rosa26-LSL-EYFP	YFP	cell nuclei/membrane /cytoplasm	Pinsonneault et al. 2023

#### Analysis of MG proliferation during the reprogramming process

The proliferation of MG is a pivotal step in MG reprogramming. In zebrafish, progenitor cells originating from MG rapidly multiply and differentiate into neurons, ultimately restoring retinal function. A similar pattern is observed in mice, making an evaluation of MG cells’ proliferation potential essential for a thorough understanding of MG reprogramming (Gao et al. 2022).

Common cell cycle markers include PCNA, Mki67, Phospho-histone H3, cyclin family proteins, Bromodeoxyuridine (BrdU), and 5-ethynyl-2’-deoxyuridine (EdU). PCNA serves as a co-factor for DNA polymerase during the S phase of the cell cycle (Kurki 1986), while Mki67 is expressed throughout the cell cycle, except during the resting phase and early G1-phase (Zacchetti et al. 2003). Phospho-histone H3 is used to label mitotic cells and nuclei (Chippalkatti and Suter 2021). BrdU and EdU, thymidine analogs, are widely employed to explore S-phase progression (Chippalkatti and Suter 2021). However, BrdU is known for its cytotoxic and teratogenic properties (Taupin 2007), and has been shown to inhibit astrocyte differentiation (Wang et al. 2022), warranting caution in its use during MG reprogramming. Unlike BrdU, EdU does not exhibit these drawbacks (Ning et al. 2013).

The method of multiple labeling involves sequential use of different markers to identify cell cycle stages, facilitating the distinction of cell division timing, states, and patterns (Solius et al. 2021). In a study involving ShH10-GFAP-β-catenin injection, Yao et al. utilized a double-labeling approach with EdU and BrdU to examine proliferating MG, finding that only a small fraction was labeled, indicating most undergo a single cell division (Yao et al. 2018). In zebrafish, chick, and mice, Hoang et al. employed PCNA and EdU to label proliferating MG but lacked evidence for subsequent trans-differentiation into neurons or progenitor cells (Hoang et al. 2020). Campbell et al. used EdU to show that cannabinoid signaling, Midkine, fatty acid-binding proteins, and fatty acid synthase enhance de-differentiation and proliferation of MG in mouse or avian retinas (Campbell et al. 2021a, b, c).

Interestingly, proliferation is not always required for MG reprogramming, as MG can be directly reprogrammed into various retinal cell types without proliferating (Todd et al. 2021, 2022; Zhou et al. 2020; Pinsonneault et al. 2023; Xu et al. 2023). Compared to direct reprogramming, the indirect approach through proliferation ensures MG cells are not depleted, favoring retinal repair. The challenge lies in regulating proliferation to avoid risks like tumorigenesis. Additionally, it poses the intriguing question of whether dividing MG cells are more amenable to neuronal reprogramming.

### Molecular analysis of MG-derived neurons during the reprogramming process

To confirm MG reprogramming, it is crucial to establish the existence of an intermediate phase marked by the simultaneous expression of MG and neuron-specific markers. An in-depth examination of transcriptional, epigenetic, and metabolic levels has been conducted to identify particular molecular features within cell groups.

#### Capture the transcriptional changes of MG by scRNA-seq

The application of scRNA-seq permits examination of gene expression at the single-cell level. This technology is used to investigate MG reprogramming across various domains, such as analyzing the heterogeneity of MG reprogramming capability, exploring the gene regulatory networks linked to MG reprogramming, evaluating interactions between MG and other retinal cells, as well as characterizing intermediate states and end-cell identities.

Previous research has underscored the heterogeneity in MG structure, distribution, morphology, and responses to retinal injury across species (Graca et al. 2018). For example, scRNA-seq has unveiled the heterogeneity of quiescent MG in different dorsal, central, and ventral retina regions in zebrafish (Krylov et al. 2023). Moreover, Celotto et al. used scRNA-seq to map MG differentiation pathways during zebrafish retina regeneration (Celotto et al. 2023). This comprehensive analysis provided by scRNA-seq has opened pathways for studying MG heterogeneity in mammals. In human samples, Menon et al. identified three distinct subpopulations associated with MG function, age-related macular degeneration, and iron homeostasis (Gautam et al. 2021). However, reports on the heterogeneity of quiescent or activated MG in mice are currently lacking, highlighting the need for further exploration. Takahiro Masuda et al. employed a combination of single-cell analysis techniques, including single-molecule fluorescence in situ hybridization, immunohistochemistry, and computational modeling, to comprehensively characterize microglia subgroups that varied over time and region (Masuda et al. 2019). This methodology could potentially extend to studying MG heterogeneity in mice, revealing subtypes relevant to MG reprogramming.

Hoang et al. identified extensive gene regulatory networks that guide vertebrate retina regeneration using scRNA-seq (Hoang et al. 2020). This technique also exposed several crucial regulators of MG reprogramming in mice, such as Midkine (Campbell et al. 2021a, b, c), NFI (Hoang et al. 2020), NF-kB signaling (Palazzo et al. 2022), Cannabinoid signaling (Campbell et al. 2021a, b, c), Fatty acid-binding proteins and fatty acid synthase (Campbell et al. 2021a, b, c), Polycomb repressor complex 2, and Enhancer of Zeste 2 (Campbell et al. 2022). Guo et al. provided single-cell resolution data regarding the cell fate continuum during induced pluripotent stem cell reprogramming. By creating single-cell orientation tracking as a novel analytical tool, they discovered previously unknown bifurcation points along the reprogramming process and proposed a general bifurcation model for cell fate decisions (Guo et al. 2019). Zhou et al. developed a “cell fate index” to assess cell fate conversion at critical “decision points”, where cells either reprogram or revert to their original states. These methodologies could be utilized to probe MG reprogramming processes (Zhou et al. 2019). Additionally, investigating the molecular traits of reprogrammed MG can lead to identifying promising targets for MG reprogramming, thereby enhancing its efficacy. For example, Pten deletion in RGCs has been shown to stimulate significant optic nerve regeneration. The Smart-seq2 single-cell sequencing approach identified further pro-regeneration genes downstream of Pten deletion. Furthermore, in a mouse glaucoma model, genes such as Anxa2 and Mpp1 demonstrated notable neuroprotection and visual function preservation (Li et al. 2022).

Ultimately, scRNA-seq is instrumental in the definitive validation of MG-derived retinal neurons by offering insights into their molecular characteristics. Through trajectory analysis using scRNA-seq, Hoang et al. and Jorstad et al. demonstrated that MG-derived precursors evolved into bipolar and amacrine-like cells (Hoang et al. 2020; Jorstad et al. 2017).

#### Capture epigenetic changes of MG by ATAC-seq

MG reprogramming necessitates manipulation of their epigenetic age, a process that includes the establishment, maintenance, or reversal of epigenetic properties to remove differentiated phenotypes and promote pluripotency (Wang et al. 2017; Basu and Tiwari 2021).

ATAC-seq is a technique used to explore chromatin accessibility (Chen et al. 2018). Hoang et al. employed ATAC-seq to identify differentially accessible chromatin regions in zebrafish and mice after injury, revealing species-specific changes and accessibility footprints. Their research found evolutionarily conserved gene regulatory networks that govern temporal patterning, neurogenesis, and cell-fate specification across key cell types in the developing mouse retina by integrating gene expression data with ATAC-seq at the single-cell level (Lyu et al. 2021).

Jorstad et al. demonstrated that adult MG can generate neurons using a histone deacetylase inhibitor and MG-specific Ascl1 overexpression. High throughput sequencing of ATAC-seq results indicated that the inhibitor increased accessibility at crucial gene loci in MG, facilitating reprogramming. Additionally, their research showed that Jak/Stat3 signaling impacts Ascl1 chromatin binding and inhibits neuronal regeneration from MG in the adult mouse retina, while promoting DNA demethylation in the later stages enhances MG reprogramming (Jorstad et al. 2020).

### Confirming the function of MG-derived neurons at the terminal stage of MG reprogramming

The ultimate aim of MG reprogramming is to restore visual function, emphasizing the need to confirm the successful incorporation of MG-derived cells. It is crucial to compare their morphology and molecular changes to normal retinal neurons and conduct comprehensive electrophysiological assays and functional tests.

Jorstad et al. stained for the presynaptic ribbon marker Ctbp2 and postsynaptic marker PSD95 to assess whether MG-derived neurons form synaptic connections with host circuitry. They also used serial block-face scanning electron microscopy to rebuild MG-derived cell processes and visualize potential synaptic contacts with higher precision. Their findings showed that MG-derived neurons interacted with host cone terminals in a manner similar to horizontal and bipolar cells (Jorstad et al. 2017).

**Fig. 3:**
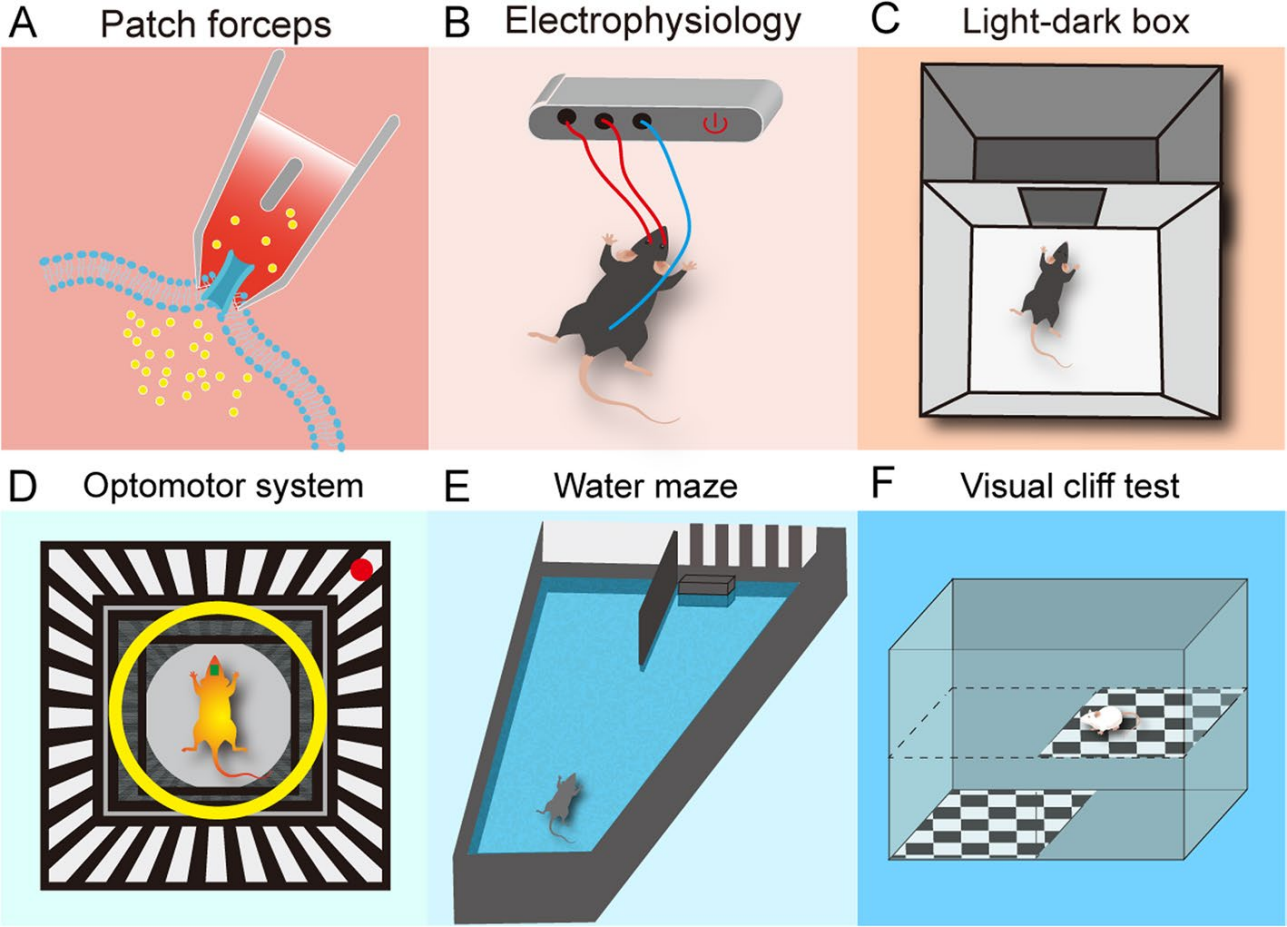
The tools used for testing the neural function of MG-derived retinal neurons. **A** A patch clamp was used to confirm the function of MG-derived neurons at the cellular level. **B** Electrophysiological technique was used to confirm the function of MG-derived neurons at the tissular level. **C-F** Light/dark transition test (**C**), optomotor system (**D**), water maze (**E**), and visual cliff test (**F**) were used to confirm the function of MG-derived neurons at the individual level

Whole-cell electrophysiological recordings revealed that MG-derived horizontal cells, bipolar cells, and RGCs respond more swiftly and robustly to light stimulation than MG (Todd et al. 2022; Jorstad et al. 2017, 2020). The synaptic mechanisms of reprogrammed RGCs can be explored using spontaneous postsynaptic currents to determine if they have developed ionotropic glutamate receptors for excitatory inputs (Fig. 3A). RGCs will show action potentials in response to light when integrated into the retinal neural circuit (Guttenplan et al. 2020). Zhou et al. also noted ON cell spikes in response to LED light under a two-photon microscope (Zhou et al. 2020). Examining visual evoked potentials in the primary visual cortex in response to light flashes offers additional insights into the central projections of MG-derived RGCs (Hamilton et al. 2021). It is noteworthy that electrical activity in ganglion cells or nerve fibers does not affect the a- and b-waves of flash ERG, which are commonly used to assess the function of photoreceptors (a-wave) and bipolar cells (b-wave) (Heikkinen et al. 2008; Friedburg et al. 2001) (Fig. 3B). Behavioral tests such as the light/dark transition test, optokinetic response, water-maze visual discrimination tasks, and the visual cliff test are frequently used to further evaluate whether MG-to-neuron conversion could potentially restore vision. The light/dark transition test, as a passive avoidance measure, involves collecting data on time spent in darkness, distance traveled, and the frequency of movement between black and white chambers (Fig. 3C). Adjustments in the dimensions of the chamber and light exposure are necessary based on the injury model. The optokinetic response is evaluated through stimuli with defined spatial frequencies and contrasts (Fig. 3D). Visual function can also be assessed via the water-maze task (Fig. 3E), focusing on parameters like latency and distance to the platform (Prusky 2000). The visual cliff test examines stereoscopic vision by observing if the mice on the platform step towards the shallow or deep side (Wang et al. 2023a, b, c; Ji et al. 2022) (Fig. 3F).

## Conclusions

In recent years, there have been notable advancements in the methodologies for studying MG reprogramming. This domain encompasses several pivotal aspects: identifying essential genes responsible for determining cell fate, accurately targeting MG cells, and efficiently modifying gene expression patterns. Despite these developments, significant challenges still persist in each area.

Firstly, our grasp of the complex, multi-layered changes in genes, proteins, and metabolic processes throughout the MG reprogramming process remains insufficient. Numerous critical genes and molecules that dictate cell fate and behaviors are yet to be identified, necessitating further investigation. Secondly, the tools for targeting and manipulating MG cells require further improvement. While AAVs have shown potential, their specificity in targeting MG cells is often lacking. Though Cre-Loxp and other genetic manipulation techniques offer high precision, their clinical application poses considerable technical and safety concerns. Thirdly, the translation of genetic information into functional biological outcomes involves multiple phases, including transcription, translation, and post-translational modifications. The reprogramming of MG cells is not driven by changes in the expression of isolated genes, but rather by a comprehensive restructuring of the gene expression landscape. Recent studies have highlighted essential genes such as Ascl1, Ptbp1, and NFI, which offer valuable insights and suggest promising directions for future research. Lastly, one of the most challenging aspects is replacing damaged RGCs, photoreceptors, or other cell types with functionally equivalent cells derived through MG reprogramming. Achieving this objective requires overcoming numerous technical and biological obstacles.

Several researchers have offered valuable insights into selecting key genes. For instance, Hoang et al. employed bulk RNA-seq, scRNA-seq, and ATAC sequencing to assess changes in MG gene expression patterns following injury across different species (Hoang et al. 2020). Likewise, Yin et al. used bulk RNA-seq and scRNA-seq to identify alterations in the MG gene expression profile after sodium iodate-induced injury (Yin et al. 2024). The application of omics techniques by these researchers has significantly expanded the pool of potential key genes for MG reprogramming. In recent years, the resolution and accuracy of single-cell omics have steadily improved, facilitating a deeper analysis of cellular changes at multiple levels. Employing these advanced technologies, similar analytical approaches could uncover critical genetic networks that impede MG reprogramming in mammals.

Regarding the precise targeting of MG cells, several enhancements are being explored. The efficacy of AAV in specifically targeting MG cells has come under scrutiny due to issues with fluorescence leakage. To date, 13 primates and over 100 non-primate AAV serotypes have been identified. As new serotypes emerge, more effective targeting options are likely to be developed. Additionally, novel methods for modifying AAV capsid proteins are beginning to surface. These modifications yield AAVs with reduced immunogenicity and enhanced targeting capabilities (Büning and Srivastava 2019). Given the potential of AAVs in clinical settings, it would be premature to dismiss AAV-mediated genetic manipulation entirely. In addition to the widely utilized MG manipulation tools, AAV and Cre-Loxp mouse lines, chemical reprogramming offers distinct clinical advantages and has demonstrated promising outcomes in MG cell reprogramming. For instance, Zhang et al. successfully achieved the transdifferentiation of MG cells into RGCs using MAP4Ks inhibitors (Zhang et al. 2023). CRISPR technology, known for its robust gene-editing capabilities, has also been employed for MG cell reprogramming (Zhou 2020). Various gene delivery methods, such as virus-like particles, nanoparticles, and extracellular vesicles, can meet a wide range of delivery needs and hold great potential as tools for MG cell reprogramming (Table 3).

**Table 3:** The advantages and disadvantages in current and potential tools for manipulating MG cells in vivo

	Technologies	Advantages	Disadvantages	References
Current tools	AAV and its derivatives	1. Low Immunogenicity 2. Broad Tissue Specificity 3. Long-term Stable Gene Expression 4. Relative Safe	1. Limited Gene Loading Capacity 2. Immune Response 3. Low Delivery Efficiency 4. Potential Repeated Dosing Issues	Akil 2020; Shchaslyvyi et al. 2023; Xu et al. 2021
	Cre-Loxp system	1. High Efficiency 2. Spatial and Temporal Specificity 3. Flexible 4. Proven Reliability	1. Potential Off-Target Recombination 2. Toxicity of Cre Enzyme 3. Limitations of LoxP Sites 4. Background Activity	Metzger et al. 1995; He et al. 2017; Vooijs et al. 2001; Song and Palmiter 2018
	Chemical Small Molecule	1. Avoidance of Gene Mutations and Tumor Risk 2. Easy to Implement 3. Precise Control of Cell Fate 4. Avoidance of Ethical	1. Unclear Long-Term Effects 2. Unclear Reprogramming Efficiency and Stability 3. Potential Cytotoxicity 4. Complex Screening Process	Li et al. 2013; Liuyang et al. 2023; Wang et al. 2023a, b, c
	CRISPR/Cas9 and its derivatives	1. High Specificity and High Efficiency 2. Flexible 3. Multiplex Gene Editing 4. Reversibility	1. Off-Target Effects 2. Unclear Long-Term Effects 3. Unpredictability of Gene Editing 4. Technical Complexity	Chen et al. 2014; Fu et al. 2013; Sander and Joung 2014; Wang et al. 2016
Potential tools	Virus-Like Particles	1. Relative Safe 2. High Specificity and High Efficiency 3. Flexible	1. High Manufacturing Costs. 2. Complex Production Processes.	Raguram et al. 2022; Taghizadeh et al. 2024
	Nanoparticles and their derivatives	1. Low immunogenicity	1. Lower gene delivery efficiency 2. Potential cytotoxicity	Parat et al. 2015
	Extracellular Vesicles	1. Low immunogenicity 2. High Efficiency	1. Higher Manufacturing Costs. 2. Complex Production Processes	Thankam et al. 2023

The methods for modifying gene expression patterns have become increasingly diverse in recent years. Instead of altering the DNA sequence itself, epigenetic editing adjusts the chemical structure surrounding DNA to regulate gene expression. This approach reduces the genetic risks associated with gene editing as it does not change the genomic information (Cappelluti et al. 2024). When coupled with gene knock-in technology, STE Mining can enhance the expression of multiple genes in a single promoter knock-in (Yao et al. 2024). The CRISPR-Cas12a system allows for the simultaneous editing of multiple genes (Campa et al. 2019). Continued application of emerging technologies for multi-gene editing could lead to better reprogramming outcomes for MG reprogramming.

Several critical questions remain unanswered. What are the specific criteria and standards for reprogramming in mice? Can MG reprogramming be fully realized in mammalian retinas? How can we effectively manipulate MG using multiple approaches simultaneously? Is it feasible to employ retinal organoids to investigate MG reprogramming? Delving deeper into these questions and developing more effective technologies for manipulating MG, based on a comprehensive understanding of the barriers to MG reprogramming in mammalian retinas, is essential.

In conclusion, MG reprogramming holds promise as a therapeutic approach for blinding conditions such as glaucoma and retinitis pigmentosa. However, effective MG reprogramming remains elusive. As advanced technologies emerge, the challenges that have hindered progress in MG reprogramming may be overcome, ultimately benefiting more blind patients.

## Data Availability

The original images of Figs. 2A and B are available from the corresponding author upon request. Other data generated or analyzed during this study are included in existing literature supplementary information files.

## Abbreviations

MG: Müller glia
AAV: Adeno-associated viruses
scRNA-seq: Single-cell RNA sequencing
ATAC-seq: Assay for Targeting Accessible-Chromatin sequencing
RGCs: Retinal ganglion cells
shRNA: Short hairpin RNA
sgRNA: Small guide RNA
BrdU: Bromodeoxyuridine
EdU: 5-ethynyl-2’-deoxyuridine
